# Frontoethmoidal encephalocele as a cause of recurrent meningitis

**DOI:** 10.1590/0037-8682-0236-2023

**Published:** 2023-07-24

**Authors:** Melis Deniz, Feyza Kabar, Atakan Besnek

**Affiliations:** 1 Sanlıurfa Training and Research Hospital, Department of Pediatric Infectious Diseases, Sanlıurfa, Turkey. Sanlıurfa Training and Research Hospital Department of Pediatric Infectious Diseases Sanlıurfa Turkey; 2 Sanlıurfa Training and Research Hospital, Department of Pediatric Radiology, Sanlıurfa, Turkey. Sanlıurfa Training and Research Hospital Department of Pediatric Radiology Sanlıurfa Turkey; 3 Sanlıurfa Training and Research Hospital, Department of Neurosurgery, Sanlıurfa, Turkey. Sanlıurfa Training and Research Hospital Department of Neurosurgery Sanlıurfa Turkey

**FIGURE 1:**
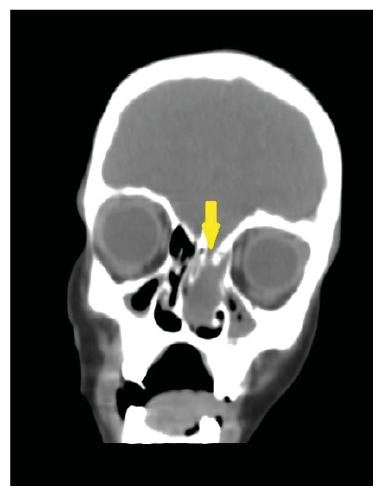
On coronal reformated CT images, a bony defect is seen on left lamina cribrosa. (yellow arrow). Heterogenous image of encephalocele and mucosal tissue is seen on the left ethmoid sinus and nasal cavity.

**FIGURE 2:**
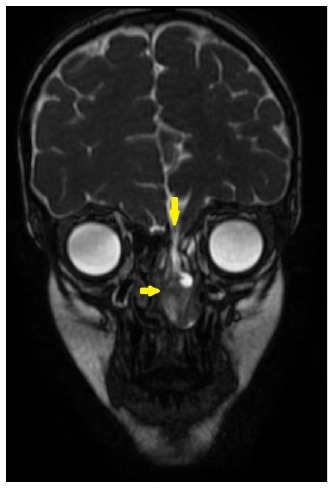
Coronal balanced steady-state gradient echo sequence image shows the defect on left skull base. Encephalocele is seen on left ethmoid sinus and nasal cavity, which is connected intracranially through the defect. Deviation of the nasal septum is also noted (yellow arrows).

**FIGURE 3:**
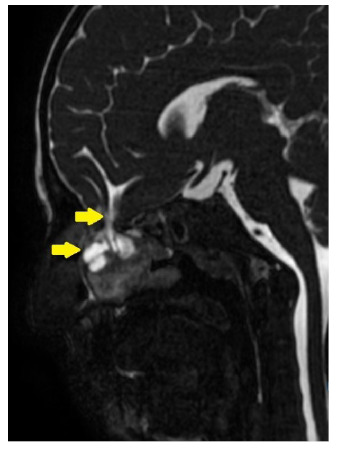
Sagital balanced steady-state gradient echo sequence image shows the defect on left skull base. Encephalocele is seen on left ethmoid sinus and nasal cavity which is connected intracranially through the defect (yellow arrows).

A 9-year-old boy with fever and headache was admitted to the pediatric infectious disease ward. Upon admission, the patient presented with nuchal rigidity and positive Kernig-Brudzinski signs. Serum levels of inflammatory markers were also elevated. Cerebrospinal fluid (CSF) analysis showed CSF pleocytosis, elevated CSF protein level (448 mg/dL), and decreased CSF glucose level (8 mg/dL), suggestive of bacterial meningitis. Blood and CSF cultures were negative. Polymerase chain reaction (PCR) of the CSF revealed *Streptococcus pneumoniae* as the causative agent. Vancomycin-ceftriaxone combination therapy was initiated. Based on the patient’s history of two episodes of pneumococcal meningitis, a detailed immunological analysis was performed and found to be normal, excluding immune deficiency. Cranial computed tomography (CT) was performed because cranial skeletal defects can cause recurrent meningitis. The cranial CT revealed a bony defect in the left lamina cribrosa (Figure 1). For a detailed analysis, cranial magnetic resonance imaging (MRI) was performed, which confirmed a defect at the base of the left skull. An encephalocele was observed in the left ethmoid sinus and nasal cavity, connected intracranially through the defect (Figures 2-3). These lesions may have led to the transmission of bacteria from the nasal cavity to the meningeal space. The patient underwent surgery after completion of antibiotic therapy.

Frontoethmoidal encephalocele is a protrusion of intracranial contents through a defect in the joint between the frontal and ethmoidal bones, directly connecting the brain with the external region, leading to pathogenic invasion^1^^,^^2^. The integration of cranial CT with MRI is recommended for diagnosis and surgical guidance^2^^,^^3^. CT is useful for observing deformities in the craniofacial bones and the site and dimensions of the lesion^3^. MRI helps analyze the internal structure of the sac and highlights unusual features of the brain^3^. Delayed diagnosis may result in recurrent meningitis^1^. Further, prompt surgical management is necessary^2^^,^^3^. 
